# Some Medical Aphorisms and Experiences

**Published:** 1911-07-08

**Authors:** Seymour Taylor


### Hospital Clinics.

### SOME MEDICAL APHORISMS AND EXPERIENCES.

By SEYMOUR TAYLOR, M.D., F.R.C.P.

(A Lecture given at the West London Post-Graduate College.)

The subject of my lecture to-day is named "Some Medical Aphorisms," and although I may be considered a plagiarist in the selection of the title, I must ask you to acquit me of any charge of attempting to copy the work of a well-known hospital physician, Dr. Gee. Many of his dicta are absolutely correct, though there are some which do not meet with the endorsement of every clinician. And probably the same observation will be made of some of the statements which I am about to submit to you.

An aphorism is described as a maxim, or a general rule. I would prefer to select the latter definition, since in medicine very few axioms obtain. Medicine was never an exact science, and never will be, so long as the varying eddies of life and its environment are manifest. So all that a medical observer can say is that, in his experience, certain signs, symptoms and data are in the main correct; and the more observant he is, and the greater the opportunities of observation which he has enjoyed, the more likely is he to be correct in the deductions which he submits to his audience.

In placing before you the following clinical suggestions or intimations for your acceptance, I think it best and most convenient to arrange them in some order of anatomical sequence, though some of them may overlap.

Let me therefore begin with the

### Digestive Tract.

i. The site of abdominal pain is by no means always a guide to the seat of lesion. I remember, among many others, a patient in whom the situation of pain, tenderness, together with other symptoms, and the history of illness pointed to volvulus of the descending colon. When the belly was opened by a surgical colleague, he found a diseased and perforated appendix.

ii. In all cases of abdominal trouble in which the diagnosis is obscure, the surgeon's incision, if laparotomy be decided upon, should be such as will command the appendix.

iii. Cancer of the bowel is a chronic disease, but may exist for many weeks before it betrays itself, and then often the first symptoms are those of acute obstruction.

iv. In malignant disease of the abdomen, the end often comes with startling suddenness, although the patient may have appeared to have been no worse on the day preceding his or her death.

v. The examination of a patient who has gallstones or in whom the presence of gallstones is suspected, will often provoke an attack of colic a few hours after examination, the calculi being disturbed or dislodged by the process of manipulation.

vi. In biliary colic, olive oil in large doses is only useful when the calculi are numerous and small. It is useless when there is evidence that a large calculus, anything over the size of a horse-bean, is present.

vii. In all cases of gastric disturbances or of colic, no matter whether the pain be referred to stomach, to intestine, to the liver, or to the kidney, never omit to inquire into the presence or absence of the knee jerks.

viii. Copaiba is one of our best remedies in cases of portal dropsy.

### Respiratory Tract.

i. Micrococci will produce entirely different results according to the site of their invasion. For example, the pneumococcus may remain in the buccal cavities for weeks, and probably longer; but when it invades the alveoli of the lungs, or the pleura, pneumonia results in the former case, and suppuration (empyæma) in the latter.

ii. The lung is not the only organ or tissue which is liable to pneumococcal infection, and the lethal effects of the pneumococcus may be equally pronounced when it invades the accessory passages of the air tract (*e.g.* antrum) as when it involves the lung.

iii. Tuberculous phthisis in elderly people between sixty and seventy years old is by no means uncommon, and often two or more members of the same family are affected at about the same age.

iv. In chronic bronchitis death is most frequently due to the supervention of an acute attack.

v. The chronic bronchitic should always be a spare eater, and should abstain from alcohol. The recurrences of acute attacks are considerably lessened thereby, and the general health and comfort of the patient considerably enhanced.

vi. The phenomenon known as Cheyne-Stokes respiration is of the gravest omen. I have not known any patient recover in whom this symptom has been present.

vii. The large mucous râle, when it comes on during an acute affection of the respiratory tract, is one of solemn prognosis. I have only known a few recover in whom it occurred.

viii. In pleural effusion, if aspiration be decided on, it is best to drain the cavity as completely as possible. The advent of persistent short cough is the best guide to the operator that the needle should be withdrawn.

ix. In pneumonia the pulse tension is always low.

x. The death-rate from pneumonia is not so much influenced by the area of pulmonary tissue which is involved as by the virulence of the toxæmia. Some people, with severe double-pneumonia, survive; others, with only a small portion of one lung affected, die.

xi. In pneumonia the earlier the crisis occurs the lower the death-rate. It should therefore be our duty to hasten, if possible, the advent of the crisis. Until we have a suitable "vaccine," I find that the old-fashioned treatment of sweating, preferably by large doses of liquor ammoniæ acetatis, is the most useful.

xii. In pneumonia venesection is often advisable, but the indications for this step are afforded by the condition of the heart rather than by that of the lung.

xiii. Immediately, and for some two or three days after the beneficent crisis of pneumonia has occurred, the evidence afforded by the pulse and the clinical thermometer is more important than that afforded by the stethoscope.

### Circulatory System.

i. The superficial cardiac dulness in health does not extend to the mid-sternal line, but stops short about ½ inch of it.

ii. Chorea in childhood is one of the many forms of rheumatism and should be treated as such. In the great majority of cases the choreic child either develops articular rheumatism, or has had articular rheumatism, or comes of a rheumatic stock.

iii. Heart disease, therefore, whether of endocardium, myocardium or of pericardium, is quite as frequently found in juvenile chorea as it is in rheumatism which affects the joints.

iv. Mitral stenosis is the most frequent cardiac sequel of acute rheumatism of childhood.

v. Statistics tell me that if mitral stenosis be discovered in adults, there is, in the vast majority of cases, a history of acute rheumatism, or chorea, having occurred in early life, say between the ages of five and fifteen.

vi. When the intermittent pulse, which is often associated with myocardial changes, suddenly becomes regular and quickened, it is not a good sign.

vii. Southey lived before his time. The drainage tubes which are associated with his name are admirable methods of reducing dropsical accumulations, but the operation of inserting them, and the subsequent dressings must be absolutely antiseptic and Listerian.

viii. In anasarca due to heart disease we should remember that the heart itself may be, and often is, in a state of œdema.

ix. You may often, with advantage, give a patient who is suffering from cardiac failure a moderate dose of opium, even though he has some albuminuria.

x. Angina pectoris is often associated with, and an attack induced by, acute dyspepsia (hyperacidity).

xi. In aneurysm of the thoracic aorta I have never seen any benefit accrue to the patient from surgical operation on the sac itself.

xii. A patient suffering from aortic regurgitation alone should not be given digitalis in any form. I have recorded some cases and have notes of many others, in whom sudden and unexpected death has occurred after taking a few doses of this drug.

xiii. In aortic regurgitation it will be found that the patient's comfort is increased, and his dyspnœa is diminished if he has, in addition, mitral regurgitation—a kind of bicuspid "safety-valve" action.

### Urinary Tract.

i. Indicanuria is found in all cases of disease of the peritoneum, and of all affections of the mucosa of the bowel. It has therefore, by its frequency, very little diagnostic value.

ii. The crystals of uric acid are especially large, with well-defined angles and borders, in glycosuria.

iii. In determining the presence of polyuria it is fallacious to rely on the evidence of frequent micturition. This may be due to irritation of the bladder. The urine should always therefore be measured.

iv. As a rule diabetics receive more benefit as hospital in-patients than as out-patients. This is due to enforced rest in bed and a strict diet, rather than to the action of drugs.

v. Sugar in small quantities is often found in the urines of patients suffering from tertiary syphilis.

vi. The patient who has chronic tubal nephritis most often dies from an acute attack supervening, (see Bronchitis).

### Nervous System.

i. I have never known a case recover in which floccitation or carphology was present. The oldest writers have mentioned the gravity of this sign.

ii. Tremors in typhoid fever are usually indicative of deep ulceration of the bowel.

iii. A knee jerk is best elicited by obliquely striking the outer edge of the extensor tendon. I know not why.

iv. Severe delirium, apart from injury to the brain, is usually associated with some specific fever, such as typhoid, typhus, pneumonia, especially of the apex; but more especially is it associated with these fevers in an alcoholic subject.

v. The power of the will is a prominent factor in recovery from an illness. The patient who determines to live frequently does so, and in old age this determination to survive to a certain date is frequently observed. For example, in the periodical division of profits, whether annual, quinquennial, or septennial, which life assurance offices effect, it is frequently noted that feeble assurers survive, by an effort, to the date of the division and die soon afterwards. Experience teaches, therefore, that in the quarter immediately following a division of profits the death rate amongst the older policy-holders is unusually high.

### Specific Fevers.

i. In typhoid fever there is an odour from the patient's body as distinctive as that observed in typhus. The smell resembles that of damp earth, or of an ill-ventilated cellar or underground vault. During convalescence from this fever there can nearly always be found a fine wrinkling of the skin, with powdery desquamation, especially over the flanks and down the upper thirds of the outer sides of the thighs. The patient's temperature charts will, in the vast majority of cases, show a range below the normal line, for some week, ten days, or even longer, during early convalescence. This is especially so if there has been a relapse, or if the initial attack was very severe.

ii. In children with any form of febrility it is well to discount the temperature by 2° F.

iii. In children recovering from a fever, and in whom a sub-febrile temperature still persists, it will often be found that convalescence is hastened by a mild dose of alcohol.

iv. In convalescence from acute rheumatism I have often observed that a recrudescence of rheumatic pains and of temperature occurs after iron, especially the tincture, has been administered with a view to correct the anæmic condition.

Skin.

i. Acne is a disease found especially in early adolescence. It is primarily due to this epoch. In women it disappears after the first pregnancy. In men it also disappears after marriage. This may be verified by many observations, notwithstanding the presence of micro-organisms in the hair follicles, as described by dermatologists.

ii. Chlorosis in the young woman is usually cured by pregnancy.

Drugs.

i. *Iodide of Potassium.*—You will obtain better effects from iodide of potassium by prescribing small doses (say, 3 to 5 gr.) for a few days, and then rapidly increasing the dose, than by giving large doses (15 to 20 gr.) at the onset.

ii. *Nux Vomica.*—In its action on the muscular coat of the intestine the tincture of nux vomica is of more use than the corresponding dose of strychnia, which is not the only active principle which the nux vomica contains.

iii. *Opium.*—Children tolerate opium and morphia better than is generally supposed. It is merely a matter of dosage. In my experience morphia in $\frac{1}{50}$ to $\frac{1}{25}$ gr. doses, every two hours during the daytime, is the finest remedy which we have for whooping cough.

iv. Sarsaparilla in the form of a decoction is a most useful adjunct to iodide of potassium in tertiary syphilis. It should be given in large doses.

v. In erythema nodosum I have found nitrate of potassium (5 gr. doses) more quickly effect a cure than any other drug.

General Experiences.

i. Rare diseases are more common in the poorer or lower ranks of society.

ii. Those people ward off disease, or being attacked, have the best chances of recovery, who can afford and enjoy a generous dietary.

iii. In all cases of malignant new growth operate early—or not at all. The disease often has an unexpectedly rapid termination, although to all appearances the patient had made no adverse progress for the past week or more.

iv. Death rarely occurs from pulmonary hæmorrhage in phthisis. When hæmorrhage is fatal, it will be found on autopsy that the ruptured vessel was aneurysmal.

v. Gastric ulcer in young women is not a primary disease. In the great majority of cases it is a sequel or climax of chlorosis. Certainly, when inquiring about antecedent ailments in young women who present signs of gastric ulcer, it will be found that they have suffered from chlorotic anæmia.

vi. An acute attack of lumbago may often be immediately relieved by passing a stout needle into the erectorspinæ mass of muscles on each side.

Such, gentlemen, are some of my experiences, and I place them before you, with all their "imperfections on my head." Possibly in the near future I may add to them in another lecture, for I have by no means exhausted them.

